# The Inhibition of KCa3.1 Channels Activity Reduces Cell Motility in Glioblastoma Derived Cancer Stem Cells

**DOI:** 10.1371/journal.pone.0047825

**Published:** 2012-10-22

**Authors:** Paola Ruggieri, Giorgio Mangino, Bernard Fioretti, Luigi Catacuzzeno, Rosa Puca, Donatella Ponti, Massimo Miscusi, Fabio Franciolini, Giuseppe Ragona, Antonella Calogero

**Affiliations:** 1 Department of Medical-surgical Sciences and Biotechnologies, University of Rome "Sapienza", Latina, Italy; 2 Department of Cellular and Environmental Biology, University of Perugia, Perugia, Italy; McGill University, Canada

## Abstract

In the present study we evaluated the expression of the intermediate conductance calcium-activated potassium (KCa3.1) channel in human glioblastoma stem-like cells (CSCs) and investigated its role in cell motility. While the KCa3.1 channel is not expressed in neuronal- and glial-derived tissues of healthy individuals, both the KCa3.1 mRNA and protein are present in the glioblastoma tumor population, and are significantly enhanced in CSCs derived from both established cell line U87MG and a primary cell line, FCN9. Consistent with these data, voltage-independent and TRAM-34 sensitive potassium currents imputable to the KCa3.1 channel were recorded in the murine GL261 cell line and several primary human glioblastoma cells lines. Moreover, a significantly higher KCa3.1 current was recorded in U87MG-CD133 positive cells as compared to the U87MG-CD133 negative subpopulation. Further, we found that the tumor cell motility is strongly associated with KCa3.1 channel expression. Blockade of the KCa3.1 channel with the specific inhibitor TRAM-34 has in fact a greater impact on the motility of CSCs (reduction of 75%), which express a high level of KCa3.1 channel, than on the FCN9 parental population (reduction of 32%), where the KCa3.1 channel is expressed at lower level. Similar results were also observed with the CSCs derived from U87MG. Because invasion of surrounding tissues is one of the main causes of treatment failure in glioblastoma, these findings can be relevant for future development of novel cancer therapeutic drugs.

## Introduction

Glioblastoma Multiforme (GBM) is the most common malignant Central Nervous System (CNS) tumor in adult, and the most difficult to cure despite the advances in surgery and adjuvant therapy [1]. It represents 30 to 60% of CNS primary tumors, with an incidence of 2 to 3 cases per 100 000 people per year [2], [3]. Only 30% of GBM patients live longer than one year after diagnosis, and the average life expectancy remains approximately 14–18 months [4], [5]. The poor prognosis for GBM patients has not improved significantly over the last decades, mostly due to the difficulties and challenges in detecting and treating this lethal cancer.

Several properties of cancer, including glioblastoma, are influenced by misregulation of ion channel expression or function [6]–[8]. Reduced expression of inward rectifier K channels [9] and increased expression of amiloride-sensitive Na channels [10], voltage-activated Cl channels [11], and BK channels [12] have been reported in several gliomas, compared to normal astrocytes. KCa3.1 channel expression may also be deregulated in glioblastoma. The KCa3.1 channel, also known as IK1, SK4, KCNN4, IKCa is a member of the calcium-activated potassium (KCa) channel family, with a unitary conductance of 20–60 pS in symmetrical 150 mMK [13], [14]. It is distinguished from the functionally related calcium-activated potassium channels of larger (100–200 pS; BK) and smaller (2–20 pS; SK) unitary conductance by its pharmacology, biophysics and physiology [13], [14]. All three family members of KCa channels were shown by Sontheimer’s group to be transcribed in glioma cells, although only BK channels were functional in the tumor, and their inhibition strongly influenced cell migration in vitro [15]. Recently our group reported the functional expression of the KCa3.1 channel in glioblastoma cell lines and demonstrated that these channels have profound effects in promoting cell migration, as shown by transwell migration assay in presence of specific KCa3.1 channel blockers [16]. Subsequently, the expression and functional activity of the KCa3.1 channel was firmly established in two glioma cell lines and in cells from one primary culture [17].

Recent evidence suggests that glioblastomas originate from a pool of stem-like cells that share properties in common with neuronal stem cells. Stemness behavior and migratory ability are closely associated and regulated by common signaling pathways [18]. Based on these data we set out to investigate whether the KCa3.1 channels are involved in the migratory process of stem-like cells isolated from tumor derived primary and permanent cell lines. We found a pronounced expression of actively functional KCa3.1 channels in the enriched fraction of cells with stem-like properties and that their selective blockage dramatically inhibited cellular motility.

## Results

### Functional KCa3.1 Channels are Expressed in the U87MG and GL261 Cell Lines

**Figure 1:**
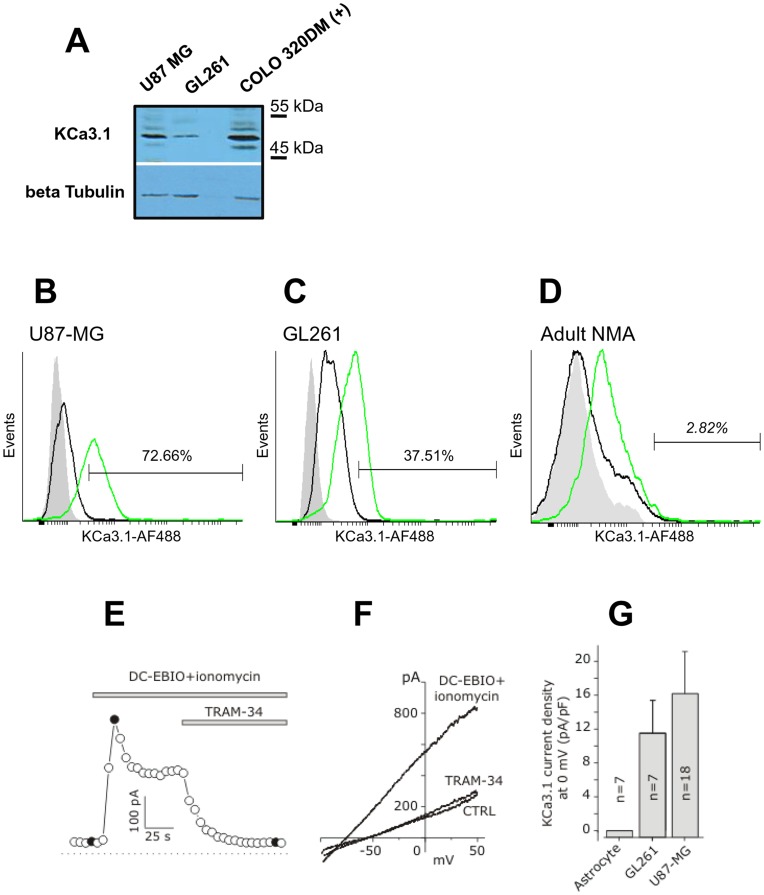
Functional KCa3.1 channels are expressed in U87MG and GL261 cell lines. (**A**) Immunoblot analysis of U87MG and GL261 showed an expression of KCa3.1 channels correlated to transcript levels. (**B**) (**C**) Cytofluorimetric analysis of KCa3.1 on U87MG, GL261 and mouse normal adult astrocytes (NMA). Cells were incubated with anti-KCa3.1 followed by AlexaFluor488-conjugated Goat anti-Rabbit antibody as reported in the Materials and methods section. Ten thousand events were recorded and analyzed with Cyflogic. Gray histograms: cellular autofluorescence; black histograms: AlexaFluor488-conjugated Goat anti-Rabbit alone; green histograms: anti-KCa3.1 (**D**) Typical time course of the KCa3.1 current from a GL261 cell, recorded from I-V curves at 0 mV, in control conditions, after application of DC-EBIO (100 µM) + ionomycin (500 nM), and following application of 3 µM TRAM-34 in the continuous presence of DC-EBIO+ionomycin. Voltage ramps were applied every 5 s. Filled circles are data points obtained immediately before the I-V curves shown in panel E. (**E**) Representative I-V curves obtained by applying voltage ramps from −100 to +50 mV from a holding potential of 0 mV, in control conditions (CTRL), following the application of DC-EBIO+ionomycin, and after addition of TRAM-34 in the continuous presence of DC-EBIO+ionomycin. (**F**) Mean KCa3.1 current density measured in mouse NMA, as control, in GL261 and U87MG glioblastoma cell lines at 0 mV, assessed as the difference between the peak current density in DC-EBIO+ionomycin and the residual current following the addition of TRAM-34 (cf. filled circles in panel **D**).

In order to determine the levels of KCa3.1 mRNA in glioblastoma cancer cells, we measured their expression by Real-time PCR on two well characterized cell lines, the human U87MG and the murine GL261. *KCa3.1* mRNA is clearly detected in both cell lines and expressed at higher levels compared to human and murine normal astrocytes. Their levels were 118.47±14.6 times higher in the U87MG and 76.13±16.52 in GL261 cells (data not shown). Western blot analysis of whole-cell lysates, performed to assess the protein expression, showed a band of ∼48 kDa in both cell lines co-migrating with the positive control provided by the specific antibody producer (Figure 1A). The amount of KCa3.1 protein detected in the U87MG is clearly higher compared to that observed from the GL261 cell line. Optical density (OD) measurements of band intensity, after normalization, estimated the KCa3.1 level in the U87MG to be about 4 times higher than in the GL261. We also evaluated the frequency of positive cells in the two cell lines by cytometric analysis (Figure 1B, C). The percentage of KCa3.1 positive cells was 72.66% in U87MG cell line and 37.51% in GL261 cell line. These frequencies are relatively high if compared to that of KCa3.1 positive cells (2.82%) found in mouse normal adult astrocytes, taken as control cells (Figure 1D).

The functional expression of KCa3.1 channels in U87MG and GL261 glioblastoma cells was verified with patch-clamp measurements in the whole-cell perforated configuration. TEA (3 mM) and octanole (1 mM) were present in all solutions to block the BK and gap junctional channels, usually co-expressed with KCa3.1 channels in glioblastoma cells (see Methods, [16]). A typical experiment illustrating the protocol used to assess the KCa3.1 current is shown in Figure 1E and F. Cells were repeatedly stimulated with voltage ramps from −100 to +50 mV from a holding potential of 0 mV, and the KCa3.1 current was assessed by first applying the KCa3.1/SK channel activator DC-EBIO (100 µM) plus ionomycin (500 nM; DC-EBIO+ionomycin), and then adding the specific KCa3.1 channel inhibitor TRAM-34 (3 µM) in the continuous presence of DC-EBIO+ionomycin. In both cell lines, following the extracellular perfusion with DC-EBIO+ionomycin, we observed the development of a voltage-independent current with a reversal potential (mean −85±3 mV for GL261 cells, n = 3, and −82±4 for U87MG cells, n = 4) close to the K equilibrium potential in our recording conditions (−90 mV). The DC-EBIO+ionomycin-induced current had in most cells an initial transient phase preceding a sustained plateau. Both the transient and sustained components could in fact be ascribed to the KCa3.1 current, as they were never observed upon application of DC-EBIO+ionomycin in TRAM-34-preincubated cells. The mean KCa3.1 current density of U87MG and GL261 cells (transient plus sustained, assessed at 0 mV) was 16.2±5.0, n = 18, and 11.5±3.9, n = 7, respectively. In contrast, no DCEBIO+ Ionomycin-activated TRAM-34 sensitive current was observed in normal mouse adult astrocytes (n = 7) (Figure 1G).

### Induction of Neurospheres and CD133 is Followed by Stimulation of the KCa3.1 Channels Expression, in the U87MG Cell Line

**Figure 2:**
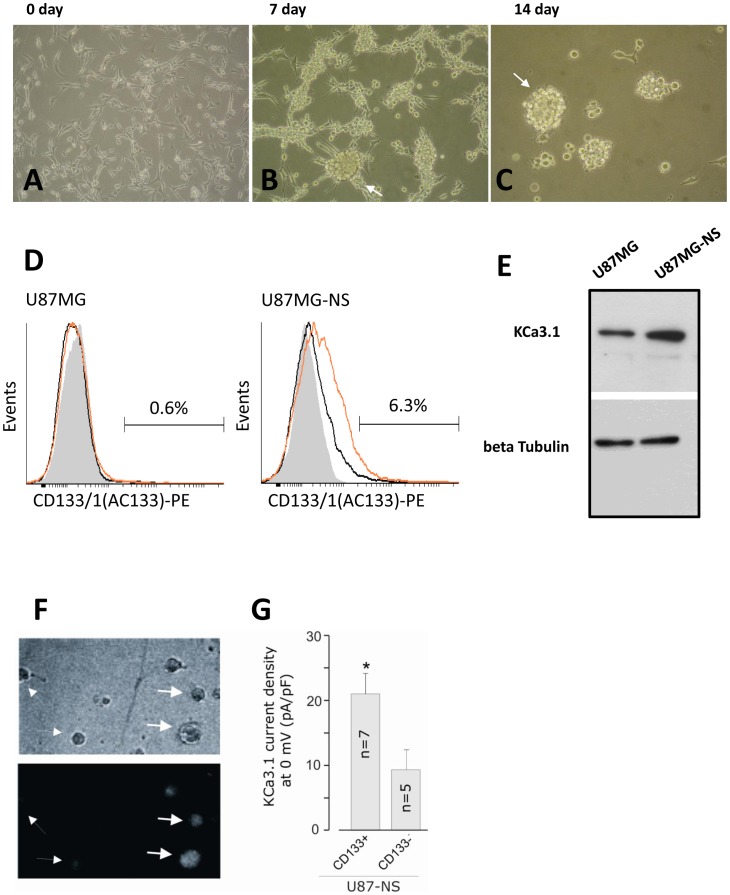
Induction of neurospheres and CD133 is followed by stimulation of the KCa3.1 channels expression, in the U87MG. (**A**) Phase microscopy image shows U87MG cells subconfluent and U87MG-derived neurosphere after 7 (**B**) and 14 days (**C**) of serum free medium conditioning. (**D**) Cytofluorimetric analysis of CD133 on U87MG and U87MG-NS. Cells were stained with PE-conjugated anti-CD133(1) or histotype matched antibodies as described in Materials and methods section. Ten thousand events were recorded and analyzed with Cyflogic. Gray histogram: cellular autofluorescence; black histogram: Isotypic control; orange histogram: anti-CD133(1) (**E**) Immunoblot analysis of U87MG and U87MG-NS showed an expression of KCa3.1 channels correlated to transcript levels. (**F**) Phase contrast (top) and immunofluorescence (bottom) images of mechanically dissociated U87MG-NS cells following 30 min incubation with the anti-CD133 antibody, showing CD133^+^ and CD133^−^ cells (indicated by thick and thin arrows, respectively). (**G**) Bar plot showing the mean KCa3.1 current density measured a 0 mV in CD133^+^ and CD133^−^ U87MG-NS cells. Experiments were performed as described in the legend of Figure 1D–F. *ANOVA test, p<0.05.

We next sought to examine the expression and function of KCa3.1 channels in U87MG cells cultured in stem cell permissive medium. Cell conditioning was assessed with optical microscopy and cytofluorimetry by verifying the appearance of neurospheres and CD133^+^ cells, which were monitored up to three weeks afterwards (Figure 2A, B, C). CD133 is a marker of most tumor stem-like cells, especially in brain tumors [19]. CD133^+^ cells cumulated to a maximum of 6.3% (Figure 2D) after 10 days of conditioning (U87MG-NS), and they were still positive after 16 days (4.6%, data not shown).

In order to verify the stem-like properties of U87MG-NS we performed Real-time PCR to examine the expression of *nestin* and *GFAP.* Changes in the expression of *nestin*, a marker of neural stem cells, and *GFAP*, a marker of differentiated astrocytes, are indicative of differentiation *in vitro* of stem-like cell derived from glioblastoma [1]. Compared to untreated U87MG, *nestin* expression was increased 2.68±0.56 times (p<0.001), whereas *GFAP* was found to decrease (0.516±0.05, p<0.05). These results were confirmed by immunofluorescence staining of the U87MG and U87MG-NS with antibodies against CD133, GFAP, and nestin (see Supplementary data).

We then examined the KCa3.1 channel expression in U87MG-NS. The expression of *KCa3.1* mRNA was 2.02±0.10 times higher than in the untreated cells (p<0.001). Also the amount of KCa3.1 protein detected in the U87MG-NS is higher than in the U87MG (the OD ratio is 1,8), as expected from the high mRNA levels (Figure 2E). The electrophysiological analysis performed on the CD133^+^ cells derived from U87MG-NS also demonstrated the higher activity of the channels in these cells. Specifically, we performed patch-clamp measurements on either CD133 negative or positive cells, after staining with anti-CD133 antibodies by immunofluorescence (Figure 2F). As shown in Figure 2G, both cells displayed KCa3.1 currents, as evaluated by applying the standard protocol in the whole-cell configuration. Notably, CD133^+^ cells were found to express a significantly higher level of KCa3.1 current density (21.3±3.7 pA/pF, n = 7, vs 8.1±3.5 pA/pF, n = 5, respectively; p<0.05).

### The Subset of CD133^+^ U87MG Cells Express Higher Levels of *KCa3.1* mRNA

**Figure 3:**
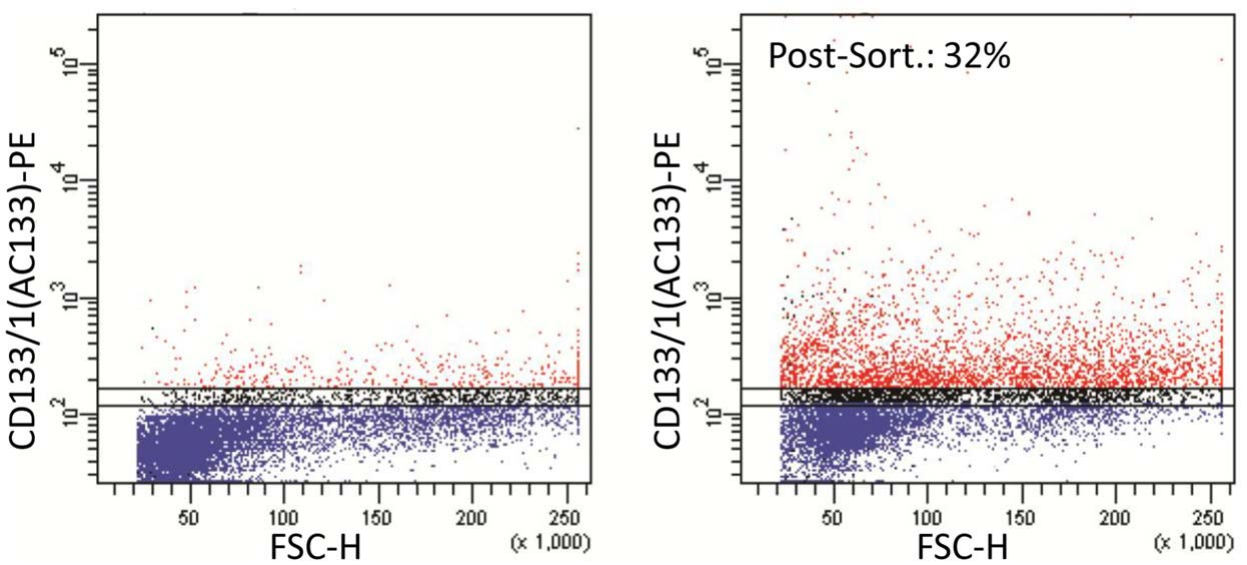
High levels of KCa3.1 mRNA channels are expressed in the subset of CD133^+^ U87 cells. Cytofluorimetric analysis of conditioned U87MG-NS were performed by staining cells with PE-conjugated anti-CD133 antibody as reported in Material and methods section. Percentage of CD133^+^ cells before (left panel) and after the sorting procedure (right panel) are shown in red. Ten thousand events were acquired and analyzed using FACs DiVa software.

Since the major focus of our studies is the *KCa3.1* channel expression in brain tumor cells with stem-like properties, we then directed our investigation towards CD133^+^ subpopulations fractionated from U87MG-NS. Using cell sorting we have obtained cell fractions with up to 32% of CD133^+^ cells (Figure 3) and with a level of *CD133* transcripts more than 11 times higher (11.63±3.23, p<0.001). *KCa3.1* mRNA level in CD133-enriched fractions, also assayed by Real-time PCR, was about 4 times higher (3.99±0.195, p<0.05) than in CD133-depleted subsets.

### The KCa3.1 Specific Inhibitor TRAM-34 Reduces the Motility of U87 MG-NS

**Figure 4:**
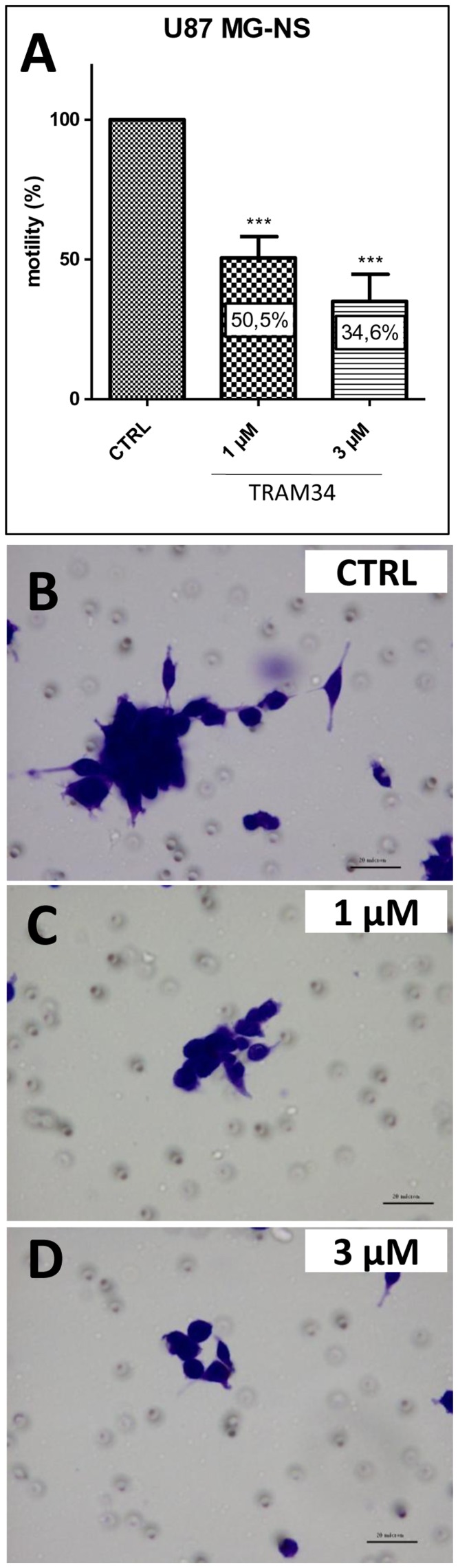
The KCa3.1 specific inhibitor TRAM-34 reduces the motility of U87MG-NS. (**A**) Quantitative analysis of cell invasion with fibronectin-coated Boyden chamber assay is described in Materials and methods section. Different concentrations of TRAM-34 (1 and 3 µM) inhibited cellular motility in a dose-dependent manner. Inhibition was statistically significant compared to untreated cells (***p<0.001). The bars are mean ± SD. (**B**, **C**, **D**) Representative microscopic fields of U87MG-NS glioblastoma stem-like cells that have migrated for 48 h through an 8 µm pore size filter in the absence (**B**) and in the presence of 1 µM (**C**) and 3 µM (**D**) TRAM-34.

We have previously shown that modulation of ion fluxes through membrane channels is essential for the stimulation of glioblastoma cell motility. Since TRAM-34 selectively inhibits ion current through the KCa3.1 channels, we tested the hypothesis that impairing ion current with TRAM-34 would have an effect on *in vitro* motility of U87MG-NS (evaluated by fibronectin-coated transwell assays). As shown in Figure 4A we found that TRAM-34 at the concentration of 1 and 3 µM inhibited motility by 49.5% ±21.52 (n = 10, p<0.001), and 65.4% ±27.46, (n = 10, p<0.001), respectively.

### KCa3.1 Channels are Absent in Adults Healthy Brain and Cerebellum, but Highly Expressed in Glioblastoma Tumor Samples and Derived Primary Cell Lines

**Figure 5:**
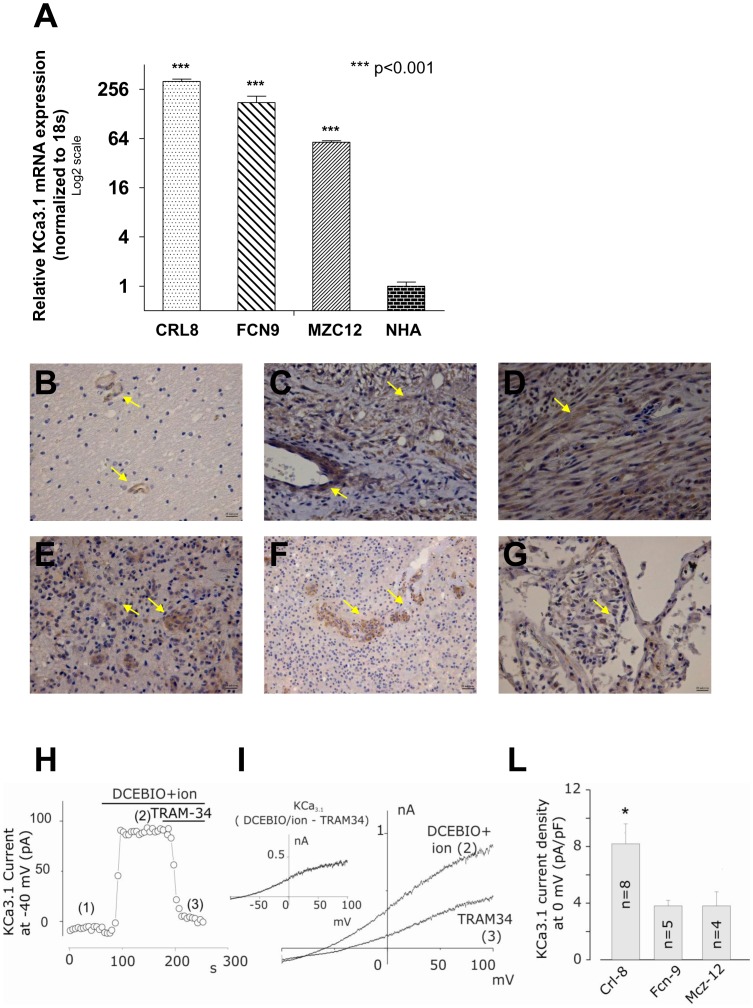
KCa3.1 channels are absent in adult’s healthy brain and cerebellum, but are highly expressed in glioblastoma tumor samples and derived primary cell lines. (**A**) Real-time PCR on three primary human cell lines of glioblastomas (CRL8, lane 1; FCN9, lane 2; MZC12, lane 3) demonstrated that KCa3.1 transcripts expression is higher compared to NHA (lane 4). (**B–G**) Immunohistochemical staining on normal human brain tissue revealed KCa3.1 protein presence only in endothelial cells of blood vessels (**B**) while we observed a diffuse staining in high grade tumors (CRL8, **C**; FCN9, **D**, MZC12, **E**) and a KCa3.1 signal only in neo-vascularisation area (glio low grade, F). As positive control we used physiological lung tissue (**G**). (**H**) Typical time course of the current recorded at −40 mV from a FCN9 cell by applying repetitive (every 5s) voltage ramps from −100 to +100 mV. 3 mM TEA and 1 mM octanole were added to block the BK and gap junctional channel, respectively, usually co-expressed with KCa3.1 channels in glioblastoma cells (21; 22). DC-EBIO (100 µM)+ ionomycin (0.5 µM) and DC-EBIO+ion +3 µM TRAM-34 were applied in succession to verify the functional expression of KCa3.1 currents (cf text). (**I**) Representative I–V relationships in presence of DC-EBIO+ionomycin, and DC-EBIO+ ionomycin +TRAM-34. Data in panel (**H**) and (**I**) are from the same experiment. *Inset*: I-V relationship of the KCa3.1 current obtained by subtracting the current ramps recorded in DC-EBIO+ion+TRAM-34 from that recorded in DC-EBIO+ion. (**L**) Plot reporting the KCa3.1 current density at 0 mV (assessed as in panel **I**) measured in the three primary glioblastoma cell lines. Since in several cells a voltage-gated K current activating at membrane potentials higher than −20 mV was present, in these cases measurements of the KCa3.1 current density were performed at −40 mV, and the assessed current density was then extrapolated at 0 mV by assuming a linear current-voltage relationship. *ANOVA test, p<0.05.

To determine whether our findings could be relevant for the study of brain tumors, we extended our investigations to the KCa3.1 channel expression of normal human astrocytes (NHA), paraffin-embedded sections from human glial tumors, and three human primary glioblastoma cell lines (CRL8, FCN9 and MZC12). The levels of KCa3.1 expression greatly differed among the investigated samples. The mean fold change relative to the NHA was 318.9±21.13 for CRL8, 176±34.64 (FCN9) and 57.6±2.4 (MZC12) (p<0.001) in the three primary cell lines (Figure 5A). Immunohistochemical staining revealed that KCa3.1 channels were absent in normal white matter brain, except for endothelial cells of blood vessels (Figure 5B), whereas they were highly expressed in sections from three different tumors (Figure 5C, D, E). A grade I astrocytoma is shown in Figure 5F with KCa3.1 positive staining confined to areas enriched in new blood vessels. In Figure 5G a tissue section from normal lung is shown as positive control.

### Functional Studies in Primary Glioblastoma Cell Lines

The functional expression of KCa3.1 channels in the three primary glioblastoma cell lines was verified with patch-clamp measurements in the whole-cell perforated configuration (Figure 5H, I, L). In all cells tested (CRL8, n = 8; FCN9, n = 5, and MZC12, n = 4) the KCa3.1 current was identified as the outward current activated by extracellular perfusion of DC-EBIO+ionomycin, and inhibited by the KCa3.1 channel-selective inhibitor TRAM-34 (3 µM) (Figure 5H, I). Figure 5L, showing the mean KCa3.1 current density assessed in the three cell lines, indicates that the CRL8 cell line has a significantly higher density than FCN9 and MZC12 cell lines. Notice that unlike stable cell lines, in primary cells the KCa3.1 currents to construct the plot have been taken at −40 mV instead of 0 mV because at this more depolarized voltage a significant voltage-gated DRK current was sometimes present that was sensibly inhibited by the KCa3.1 activating solution DC-EBIO+ionomycin. Therefore, to allow a direct comparison with the KCa3.1 current densities assessed in glioblastoma cell lines, data shown in Figure 5L are given at 0 mV. The data were obtained by linear fitting extrapolation of the I-V relationship in the −100/−40 voltage range.

### The Motility of Stem-like Cell Subpopulations Derived from Primary Cell Lines are Strongly Inhibited by TRAM-34

**Figure 6:**
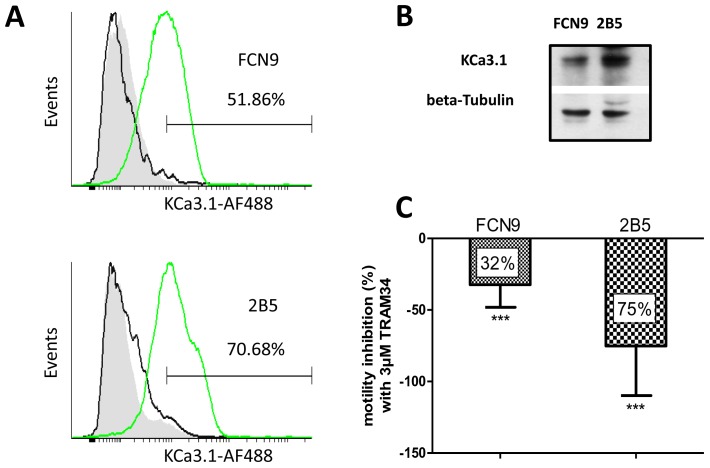
The motility of stem-like cell derived from primary cell lines, are strongly inhibited by TRAM-34. (**A**) Cytofluorimetric analysis of KCa3.1 on FCN9 (upper panel) and derived 2B5 clone (lower panel). Cells were stained with anti-KCa3.1 followed by AlexaFluor488-conjugated Goat-anti Rabbit. Ten thousand events were recorded and analyzed with Cyflogic software. Grey histograms: cellular autofluorescence; green histogram anti- KCa3.1; black histogram: AlexaFluor488-conjugated Goat-anti Rabbit. (**B**) Immunoblot analysis of FCN9 and 2B5 showed an enhanced expression of KCa3.1 channels in clonally derived subculture featuring stem-like properties (2B5). (**C**) Quantitative analysis of cell invasion performed on fibronectin-coated Boyden chamber, using 3 µM TRAM-34. Results, represented as percentage of motility inhibition compared to untreated cells, were statistically significant (***p<0.001). The bars are mean ± SD.

Last we examined the *KCa3.1* mRNA expression and the migration ability under TRAM-34 treatment in a primary cell line (FCN9) and its clonally derived subculture featuring stem-like properties (2B5) [20]. *KCa3.1* mRNA transcription and protein expression were found to be enhanced in 2B5 cells as compared to FCN9 (1.5 times ±0.2, p<0.05 for mRNA, and see Figure 6B for immunoblotting). A significant difference between the two cell lines was also found by flow cytometry (a mean fluorescent intensity of 1061.97 and 1716.37 for KCa3.1 was found in FCN9 and 2B5, respectively). The percentage of positive cells were also different between the two cell lines (51.86% in FCN9 and 70.68% in 2B5). Further, the histogram shape (i.e. an overlapping bimodal Gaussian curve in 2B5 as compared to a Gaussian curve in FCN9) reveals the presence in 2B5 cells of a subpopulation expressing high levels of KCa3.1, which is absent in FCN9 (Figure 6A). Taken together, these results indicate that the stem-like 2B5 derived clone expresses higher KCa3.1 levels than the parental FCN9 cells.

A significant reduction of motility was induced by 3 µM TRAM-34 in both cell lines. The reduction observed in 2B5 cells (75%) was however much greater than in FCN9 cells (32%; Figure 6C).

## Discussion

Along with most solid tumors consisting of heterogeneous cancer cells as well as vasculatures, stromal elements and inflammatory cells [21], GBMs display remarkable intratumoral heterogeneity and cellular hierarchy. Increasing evidence also strongly supports the concept that a subpopulation of cancer cells in the tumor mass has greater potential for cancer initiation and repopulation [22]–[27]. These cells are known as Cancer Stem Cells (CSCs) or Tumor-Initiating Cells as they share several critical properties with typical stem cells, including the capacity for self-renewal, multi-lineage differentiation, and maintained proliferation [28]–[31]. According to recent literature, glioma stem cells also promote radioresistance, tumor angiogenesis [32], [33] and drive metastasis [34]. One major problem with GBM cells is their highly infiltrative nature. As a consequence, aggressive invasion of GBM cancer cells into the normal brain tissue and spinal cord often prevents complete removal of tumor cells [35]. Migration begins when a cell responds to an external signal that leads to the polarization and extension of a “leading front” in the direction of the movement [36]. Increasing evidence shows that ion channels are necessary components of the complex machinery responsible for cell migration. Specifically, ion channels make migration possible through osmotic flows and consequent shrinking and swelling of the cell body. They are located both on the rear side of the cell and on the leading front, in which they exert also an invasive role through acidification of the ECM area and promotion of metalloproteinase proteolytic activity [37]. The infringement of homeostatic epithelium architecture, together with the acquisition of a migratory phenotype, is a key moment in tumor progression of all solid tumors. It is not yet clear whether it is mainly the stem cell component of the tumor, already displaying invasive properties in vivo, which acquires the migratory phenotype [34]. Limited knowledge is available concerning the migratory properties of glioma tumor cells in vitro in relation with KCa3.1 channel activity. The KCa3.1 channel is predominantly active in the rear edge of the cell [38] and facilitates the cellular swelling and shrinking in tumor cells during migration [37]. In addition, the KCa3.1 channel has been involved in the migratory response solicited by CXCL12, the chemokine ligand of CXCR4 [39]. Recently we have demonstrated that current density inhibition of both KCa3.1 and chloride channels in U87MG almost completely hinders migration without affecting proliferation [40]. Ion channels have been investigated in stem cells from different types of normal tissues, like KCa3.1 channels in mesenchymal stem cells derived from mouse bone marrow [41]. However, the knowledge relative to ion channels in CSCs is instead very limited [41]. This prompted us to investigate the presence and function of the KCa3.1 channel in human glioblastoma CSCs and how they relate to the mobile phenotype in these cells.

First, we show that KCa3.1 channel transcripts are expressed in different types of cultured cells such as permanently established and primary cell lines. The levels recorded by Real Time-PCR are up to 118 times higher in the U87MG, 76 times in the GL261, and 318.9, 176, and 57.6 times in three primary cell lines, compared to normal astrocytes. Lower but equally significant differences have been found between the CSCs and the parental counterpart in U87MG and the primary cell line FCN9. Differences in the fluorescence intensity of KCa3.1 channel -bound antibody and the fraction of positive cells were also observed, for the first time, by cytofluorimetry.

In the normal adult murine brain the KCa3.1 current has only been reported on activated microglia [42], brain capillary endothelial cells [43], Purkinje cells [44], and in a subpopulation of astrocytes involved in neurovascular coupling [45]–[47] [8]. Taken together, these data would suggest that in murine brain the functional expression of the KCa3.1 channel is confined to specific astrocytic cell subpopulations. Accordingly, we here report that normal adult mouse astrocytes virtually do not express KCa3.1 current. This finding is also consistent with the very low fraction (about 2.5%) of KCa3.1 positive cells found by FACS analysis in normal astrocytes. We also investigated the presence of Ca-activated K channels by immunoistochemistry in tissue sections from both human normal and tumor samples. The KCa3.1 channel presented with a diffuse and strong staining only in the samples from high grade tumors.

Channel expression allowed us to examine the question of the role of the KCa3.1 channel on the cell migratory ability by performing transwell motility assays, in presence and absence of a specific inhibitor of the channel. Striking differences were observed in the migration ability under the effect of the KCa3.1 channel inhibitor, TRAM-34. In a previous paper we found that 3 µM TRAM-34 inhibited U87MG motility by 58.5% [40]. In this work we show that the same concentration of channel blocker reduced the motility of U87MG-NS, the stem-like derived subpopulation of U87MG, by 66%.

By employing patch-clamp techniques we have also been able to probe for current activity differences between CD133 positive and negative fractions present in the U87MG-NS population. This allowed us to estimate a K current 2.6 times higher in the CD133^+^ sample. Even more striking was the reduction of 2B5 motility (−75%) under the effect of TRAM-34 compared to the reduction observed in FCN9 (−32%). The observed drop in motility well correlates with the levels of KCa3.1 channel expression in the two cell lines, due to the greater sensitivity of 2B5 to TRAM-34. The found correlation appears more significant in the light of the fact that the KCa3.1 channel is only one of a number of different types of ion channel promoting cell mobility. Overall, our results show that the KCa3.1 channel expression and function are more pronounced in the less differentiated populations, favoring a more influential role on the motility phenotype.

The highly invasive ability in vivo is a hallmark of stem cells. Moreover, it has also been hypothesized that there are two types of CSCs, one stationary and the other mobile [48]. This raises the question of whether the ion flow reduction operated by TRAM-34 alters the cell motility directly or via a signal acting upon the phenotypic switch from stationary to mobile, through a unknown regulatory pathway. This last hypothesis is attractive in view of the very recent observations showing that blocking of the plasma membrane sodium channel complex inhibits proliferation of glioma cells, in addition to migration, and that this most likely involves alterations in the gene expression program through a mechanism not yet resolved [49]. Also for the KCa3.1 channels we cannot exclude a link between modulation of current activity and gene expression reprogramming. This question remains unsolved.

## Materials and Methods

### Cell Cultures

The established mouse and human glioblastoma cell lines, (GL261 and U87MG) were grown respectively in D-MEM F-12 and D-MEM medium (Invitrogen) supplemented with 1% nonessential amino acids, 1% L-glutamine, 100 IU/ml penicillin, 100 IU/ml streptomycin and 10% fetal calf serum (FCS, Flow Laboratories) at 37°C in a 5% CO_2_ humidified atmosphere in air. Mouse and human glioblastoma cultures GL261 and U87MG were obtained from the American Type Culture Collection (ATCC, Rochville, MD, USA). Astrocytoma primary MZC12, CRL8 and FCN9 (WHO grade IV) were made in a previous work by our group from tumor specimens of patients [50]. Details of institutional committee approval and informed consent from patients are quoted in the above reference. Cell lines were cultured as described [50]–[53]. Primary mouse normal adult astrocytes were established as described [54]. 2B5 clone was derived from FCN9 subcultured cells [20]. After mechanical dissociation, single cells were resuspended in F10 medium, supplemented with 10% fetal calf serum (FCS, Life Technologies Ltd, Milano, Italy) and were plated in Petri plates (Falcon Primaria, Lincoln Park, NJ, USA). The medium was then changed every 3 days. After 14–15 days, cells were trypsinized, re-plated into 24-well plates at a density of 2,5×10^4^ cells/well and shifted into D-MEM Glutamax without serum (Life Technologies Ltd, Milano, Italy). To enrich the stem component, tumor cells were seeded at a concentration of 10^5^ cells per mL into Serum-Free medium (SFM). SFM was composed of D-MEM-F12 supplemented with human basic fibroblast growth factor (FGFb, 20 µg/L), human epidermal growth factor (EGF, 20 µg/L), B-27 Supplement (Gibco), L-glutamine, penicillin and streptomycin [1]. After the formation of primary brain tumor spheres, they were dispersed and passed in fresh medium.

### Immunohistochemistry

Immunohistochemical staining was performed with the VECTASTAIN ABC (Avidin Biotinylated enzyme Complex) system according to the manufacturer’s instructions. Briefly, the sections were air dried and fixed with ethanol. Sections were rinsed in distilled water and washed in phosphate buffer saline (PBS). After blocking sections for 30 minutes with Blocking Serum (Normal Serum), the sections were allowed to bind overnight (o/n) at 4°C using rabbit polyclonal antibody against KCa3.1 (Biosensis, No: R-1068-100) followed by 3×5 minutes washes in PBS. Biotinylated secondary antibody solution was incubated for 30 minutes at room temperature (RT). After VECTASTAIN ABC Reagent was applied for 30 minutes at RT, sections were incubated in peroxidase substrate solution until stain developed. Every section was stained for the same time-lapse. Staining was detected using Leica DM 4000B, microscopy (Germany).

### Real Time-PCR

Cellular and tissue mRNA was isolated using respectively RNeasy Mini kit (Qiagen) and the RNeasy FFPE Handbook kit (Qiagen) according to the manufacturer’s instructions. To extract RNA, cultured cells were grown in a 60-mm dish to 60–80% confluency and lysed. mRNA concentration was quantified using a DU 800 Spectrophotometer (Beckman Coulter). mRNA was converted to cDNA using the High Capacity cDNA Reverse Transcription Kit (Applied Biosystem, CA, USA) according to the manufacturer’s instructions. One µg of mRNA was used in each 20-µl cDNA synthesis reaction mix. Gene expression was quantified by a real-time PCR using the 7900HT Fast Real-Time PCR System and Power SYBR Green PCR Master Mix (both from Applied Biosystem, Warrington, UK) according to the manufacturer’s instructions and was analyzed with Biorad software. The sequences of the primers were shown in Table 1. The housekeeping gene 18S was used as internal reference. As internal control in the *KCa3.1* gene analysis we used normal mouse and human astrocytes mRNA (US Biological, MA, USA).

**Table 1:** Oligonucleotides used to perform RT-PCR.

Gene (Accession Number*)	Oligonucleotide Sequence	Amplicon lenght (bp)
hu CD133 (NM_006017.2 )	Fwd: 5′-GCATTGGCATCTTCTATGGTT-3′	170
	Rev: 5′-CGCCTTGTCCTTGGTAGTGT-3′	
hu KCa3.1 (NM_002250.2)	Fwd: 5′- ACATACTCGCAGGAAGGAGTCTCA-3′	129
	Rev: 5′-TCCACCATGGAGTTCACTTGTTCC-3′	
hu GFAP (NM_002055.4)	Fwd: 5′- CCAACCTGCAGATTCGAGA-3′	63
	Rev: 5′-TCTTGAGGTGGCCTTCTGAC-3′	
hu Nestin (NM_006617.1)	Fwd: 5′-TGCGGGCTACTGAAAAGTTC-3′	64
	Rev: 5′-TGTAGGCCCTGTTTCTCCTG-3′	
hu 18S (NR_003286.2)	Fwd: 5′-CCAGTAAGTGCGGGTCATAAGC-3′	85
	Rev: 5′-AACCATCCAATCGGTAGTAGGCG-3′	
mu KCa3.1 (NM_001163510.1)	Fwd: 5′-CACAGACACACTGTGGCTGATT-3′	177
	Rev: 5′-TTCTCCGCCTTGTTGAACTCCA-3′	
mu 18S (NR_003278.3)	Fwd: 5′-AAATCAGTTATGGTTCCTTTGGTC-3′	67
	Rev: 5′-GCTCTAGAATTACCACAGTTATCCAA-3′	

* Accession number.

### Immunofluorescence

To evaluate modulation of CD133, GFAP and nestin expression in U87MG and in U87MG-NS, cells were disaggregated and cultured o/n in 8-well chamber slide (8-well Permanox Slide, Lab-Tek, USA) precoated with 15 µg/ml ornithine (Sigma) and allowed to adhere o/n. Cells were washed once in cold PBS, fixed with 4% paraformaldehyde for 20 min, permeabilized in PBS containing 0.5% Triton X-100 (PBS-T) for 5 minutes and blocked in 2% bovine serum albumin (BSA, Sigma) in PBS-T at RT for 1 hr. The antibodies were diluited (1∶50) in 1% BSA in PBS-T and added to the chamber slide o/n at 4°C. Cells were washed 3 times in PBS-T and secondary Alexa-488 anti-goat antibody (Invitrogen, Carlsbad, CA) was added 1∶500 diluition (1% BSA in PBS-T) for 1 hr at RT. After incubation, chamber slides were washed 3 times in PBS-T, incubated 5 minutes with 4′,6-Diamidino-2-phenylindole-dihydrochloride (DAPI) (Sigma) and mounted with 90% glycerol/PBS. Negative controls were performed without the primary antibody. Cells were visualized with a Leica DM 4000B fluorescence microscope.

### Transwell Assay

Neurospheres were dispersed, harvested and resuspended in 2 ml of D-MEM-F12 with 20 µg/L of FGFb and EGF without B27 supplement and FCS and seeded at 5×10^5^ cells/well on coated fibronectin in six transwell filter chambers with 8 µm pores (Boyden chambers, BD Biosciences). D-MEM-F12 containing 2% B27 and 2% FCS was already present in the outer side of the insert. Cells were incubated at 37°C for 48 hours. After this time, we wiped the inner side of the insert with a wet swab to remove the cells while the outer side of the insert was gently rinsed with PBS, stained with 0.25% crystal violet (Sigma) for 15 minutes, rinsed again and allowed to dry. The area of the insert occupied by migrated cells was counted with a light microscope (Leica DM 4000B), by counting 10 random fields per chamber with 10X objective and ImageJ software. KCa3.1 blocker, TRAM-34, was added to D-MEM -F12 at different concentrations both in the upper and in the lower part of chamber. To perform this assay on adherent primary cells FCN9, we used the same protocol but we utilized FCS (10% in medium) as chemotactic stimulus.

### Western Blotting

Cells extracts were prepared with RIPA buffer+inhibitor cocktail, resolved on a 10% SDS-PAGE and blotted onto a PVDF membrane (Amersham HyBond-P GE Healthcare, UK). After blocking at RT in 5% BSA in PBS containing 0,1% Tween-20 for 1 hr, membranes were incubated o/n with goat antibody against KCa3.1 protein (1∶100) (Santa Cruz Biotechnology, CA) and mouse antibody against β-Tubulin (1∶1 000) (Sigma, MO) as loading control. After 3 PBS washing, membranes were incubated with anti-goat and anti-mouse horseradish peroxidase Conjugated secondary antibodies (1∶10 000) (Santa Cruz Biotechnology, CA). As positive control of immunoreactive signal was used COLO320DM cell lysate (Santa Cruz Biotechnology, CA, sc-2226). Immunocomplexes were detected by ECL Western Blotting detection system (GE Healthcare, UK).

### Electrophysiology

Macroscopic KCa3.1 currents were recorded using the perforated-patch configuration, and activated by co-application of the SK/IK activator DC-EBIO (100 µM) and ionomycin (500 nM), in the presence of 3 mM TEA and 1 mM octanole to block the BK and gap junctional currents, respectively [17], [55]. Currents and voltages were amplified with a HEKA EPC-10 amplifier, and analyzed with the PatchMaster and Origin 4.1 software. For on-line data collection, currents were filtered at 3 kHz, and sampled at 100 µsec/point. Membrane capacitance measurements were made by using the transient compensation protocol of PatchMaster.

### Solutions

For whole-cell perforated-patch recordings the extracellular solution contained (in mM): NaCl 106.5, KCl 5, CaCl_2_ 2, MgCl_2_ 2, MOPS 5, glucose 20, Na•gluconate 30, at pH 7.25, and the pipette solution contained: K_2_SO_4_ 57.5, KCl 55, MgCl_2_ 5, MOPS 10, at pH 7.20. Electrical access to the cytoplasm was achieved by adding amphotericin B (200 µM) to the pipette solution. The final access resistances were within the range of 10–20 MOhm. All chemicals used were of analytical grade. Dimethyl sulfoxide (DMSO) and TEA were from Sigma Chemical Co (St. Louis, MO, USA), and ionomycin and DC-EBIO (1-ethyl-2-benzimidazolinone) were from Tocris Cookson Ltd. (Bristol, UK). TRAM-34 was a kind gift of Dr. Heike Wulff. Stock solutions were obtained by dissolving DC-EBIO, ionomycin and amphotericin B in DMSO to concentrations of 100, 1 and 500 mM, respectively. Solutions with pharmacological agents were prepared daily at the concentrations stated, and bath-applied by a gravity-fed superfusion system at a flow rate of 2 ml/min, with complete solution exchange in the recording chamber in about 1 min. The maximal DMSO concentration in the recording solutions did not exceed 1%. Experiments were carried out at RT.

### Cytofluorimetric Analysis and Cell Sorting

Cytofluorimetric analysis of CD133 was performed using 5×10^5^ U87MG, U87MG-NS or 2B5 cells per sample. Cells were washed in PBS and resuspended in 100 µl of PBS, 30% FcR Blocking Reagent (Miltenyi Biotech). U87MG cells were incubated with a 1∶11 dilution of phycoerythrin(PE)-conjugated anti-human CD133 (clone AC133, Miltenyi Biotech) recognizing the epitope 1 [56], [57] whereas negative control was performed using isotype-matched non-specific PE-labelled antibody. KCa3.1 was evaluated by indirect staining using a 1∶100 dilution of Rabbit anti-KCa3.1 (R-1068-100, Novus Biologicals, Littleton, CO) followed by AlexaFluor488-conjugated Goat-anti-Rabbit IgG (1∶5 000 dilution of A-11034, Molecular Probe/Life Technologies, Monza, Italy). All staining were performed in PBS, 2% FCS, for 30′ at +4°C, protected from light. Cells were then washed three times in PBS and resuspended in 1 ml of PBS, 2% FCS. Sample were acquired using a FACs Aria II cytometer (Becton Dickinson) and analyzed using FACS DiVa software (v.6.1.1, Becton Dickinson), or Cyflogic software (v.1.2.1, Cyflo, Turku, Finland). For CD133^+^ cell enrichment 10^7^ U87MG-NS were stained with PE-conjugated anti-human CD133/1 as previously described and both CD133^+^ and CD133^−^ subpopulations sorted using FACs Aria II, according to CD133 expression gates.

### Statistical Analysis

Data were plotted using GraphPad Prism 5 software. Results are presented as means ± SE. Statistical analyses were performed using paired t-test and Bonferroni's one-way ANOVA post hoc test. P<0.05 was considered to be statistically significant.

## Supporting Information

Figure S1 **Evaluation of CD133, GFAP and nestin expression in U87MG and in U87MG-NS.** (**A**) Immunostaining of the U87MG (left) and the U87MG-NS (right) with antibody against CD133 (green), a most common marker of glioma-stem cells. CD133 expression increases in U87MG-NS cells, as expected. (**B**) Staining of the U87MG (left) and U87MG-NS (right) with antibody against GFAP (red), a marker of glial differentiation that decreases in glioblastoma derived cancer stem cells. (**C**) Expression of Nestin (green), a cytoskeleton protein associated with progenitor neural cells, in the U87MG (left) and the U87MG-NS (right). As expected the fluorescence signal increases in U87MG-NS. In all three of panels, the cells were counterstained with DAPI.r according to NCBI Reference Sequence.
(TIF)
