# Supplementary figures and images for: The Inhibition of KCa3.1 Channels Activity Reduces Cell Motility in Glioblastoma Derived Cancer Stem Cells
Source: PLoS One. 2012 Oct 22;7(10):e47825. doi: 10.1371/journal.pone.0047825 (PMC3478269; doi:10.1371/journal.pone.0047825)

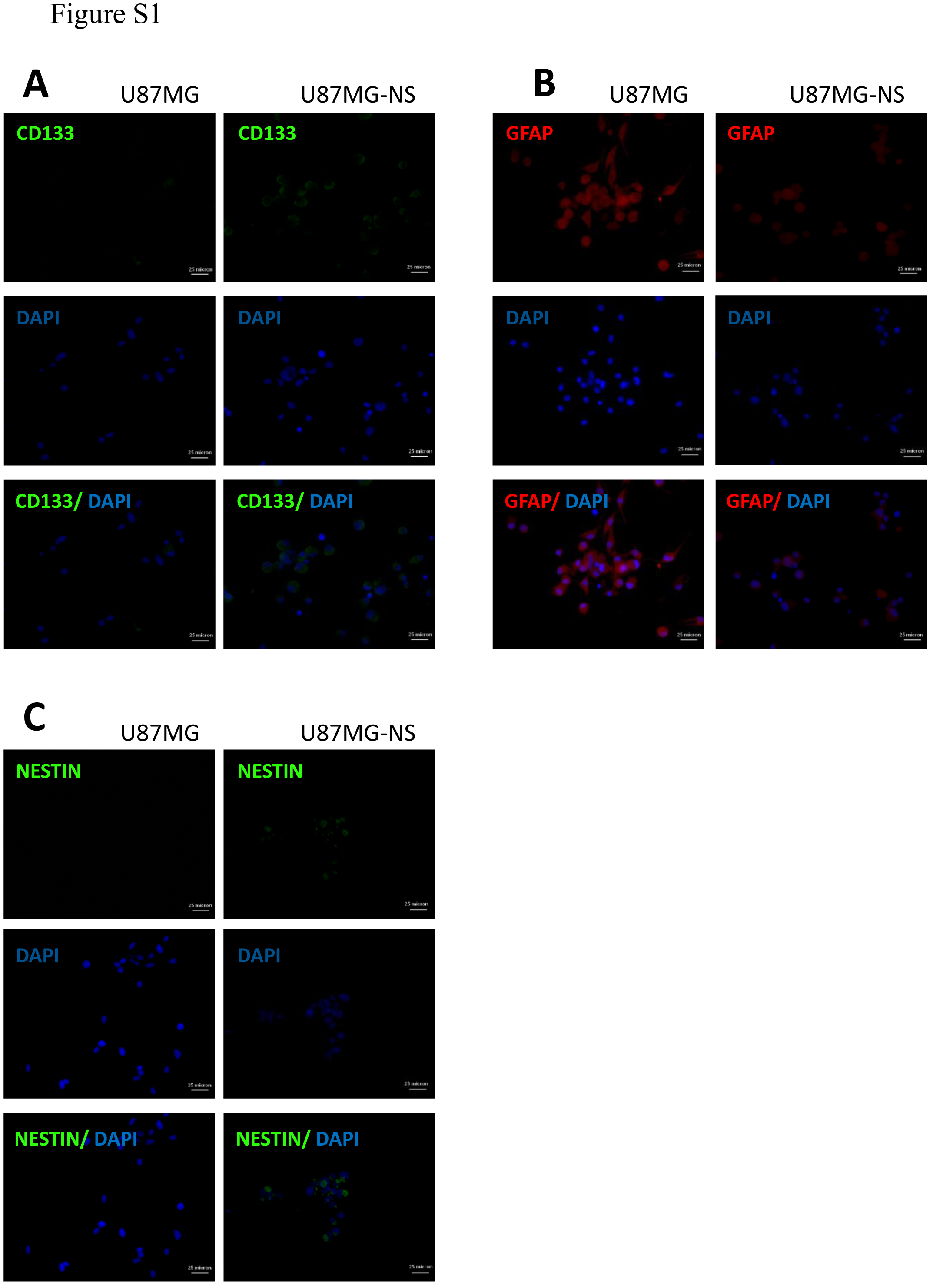

Supplement: Figure S1 — Evaluation of CD133, GFAP and nestin expression in U87MG and in U87MG-NS. (A) Immunostaining of the U87MG (left) and the U87MG-NS (right) with antibody against CD133 (green), a most common marker of glioma-stem cells. CD133 expression increases in U87MG-NS cells, as expected. (B) Staining of the U87MG (left) and U87MG-NS (right) with antibody against GFAP (red), a marker of glial differentiation that decreases in glioblastoma derived cancer stem cells. (C) Expression of Nestin (green), a cytoskeleton protein associated with progenitor neural cells, in the U87MG (left) and the U87MG-NS (right). As expected the fluorescence signal increases in U87MG-NS. In all three of panels, the cells were counterstained with DAPI.r according to NCBI Reference Sequence. (TIF) [file pone.0047825.s001.tif]
